# Ethnobotanical Description and Biological Activities of *Senna alata*

**DOI:** 10.1155/2020/2580259

**Published:** 2020-02-20

**Authors:** Oluwole Solomon Oladeji, Funmilayo Enitan Adelowo, Abimbola Peter Oluyori, Deborah Temitope Bankole

**Affiliations:** ^1^Natural Products Research Unit, Industrial Chemistry Programme, Department of Physical Sciences, Faculty of Pure and Applied Sciences, Landmark University, Omu-Aran, Nigeria; ^2^Department of Pure and Applied Chemistry, Faculty of Pure and Applied Sciences, Ladoke Akintola University of Technology, PMB 4000, Ogbomoso, Nigeria

## Abstract

*Senna alata* is a medicinal herb of Leguminosae family. It is distributed in the tropical and humid regions. The plant is traditionally used in the treatment of typhoid, diabetes, malaria, asthma, ringworms, tinea infections, scabies, blotch, herpes, and eczema. The review is aimed at unveiling the ethnobotanical description and pharmacological activities of *S. alata.* Different parts of the plant are reported in folk medicine as therapeutic substances for remediation of diverse diseases and infections. The extracts and isolated compounds displayed pronounced pharmacological activities. Display of antibacterial, antioxidant, antifungal, dermatophytic, anticancer, hepatoprotective, antilipogenic, anticonvulsant, antidiabetic, antihyperlipidemic, antimalarial, anthelmintic, and antiviral activities could be due to the array of secondary metabolites such as tannins, alkaloids, flavonoids, terpenes, anthraquinone, saponins, phenolics, cannabinoid alkaloids, 1,8-cineole, caryophyllene, limonene, *α*-selinene, *β*-caryophyllene, germacrene D, cinnamic acid, pyrazol-5-ol, methaqualone, isoquinoline, quinones, reducing sugars, steroids, and volatile oils present in different parts of the plant. The review divulges the ethnobotanical and pharmacological activities of the plant and also justifies the ethnomedical claims. The significant medicinal value of this plant necessitates a scientific adventure into the bioactive metabolites which constitute various extracts.

## 1. Introduction

The primary and safest therapeutic approach since prehistoric time is herbal medicine which has displayed significant role in primary health care development [1]. The World Health organization (WHO) appraised that 80% of inhabitants in developing countries basically rely on herbal medicines [2, 3]. Recently, it was discovered that one-third of the commonly used drugs are obtained from natural sources. This has led to the documentation of about 40,000–70,000 medicinal plant species with outstanding therapeutic potentials [4].

The scientific appraisal of the pharmacological activities of herbal plants revealed about 200,000 phytochemicals. These compounds contribute to the apparent medicinal activities displayed by plants and invariably justify the involvement of natural products in the development of novel drugs [5]. Despite these achievements, the use of herbal drugs was not universally accepted in contemporary medicine due to lack of scientific evidence and proper documentation. However, the significance of herbs in pharmacology has necessitated the provision of scientific facts on bioactive compounds and pharmacological assays of plants [6, 7].

Medicinal plants with immense biological applications have been viewed as the principal sources of therapeutic substances which could lead to novel therapeutic compounds. The search for these compounds from medicinal plants usually ends in the isolation of novel compounds and, eventually, drug developments [8]. Several medicinal plants with diverse interesting pharmacophore have been scientifically investigated, and one of these plants is *Senna alata.*

*S. alata,* also known as *Cassia alata,* is a widely distributed herb of the Leguminosae family. It is commonly known as candle bush, craw-craw plant, acapulo, ringworm bush, or ringworm plant. The plant is commonly found in Asia and Africa, and has many local names [9]. It has arrays of bioactive chemical compounds. Some of the reported chemical constituents are phenolics (rhein, chrysaphanol, kaempferol, aloeemodin, and glycosides), anthraquinones (alatinone and alatonal), fatty acids (oleic, palmitic, and linoleic acids), steroids, and terpenoids (sitosterol, stigmasterol, and campesterol) [10, 11]. These secondary metabolites are reported to display numerous biological activities [12–17].

The flower, root, leaves, seed, and bark displayed diverse biological activities [18–20]. These pharmacological activities include antimicrobial [21–23], antifungal [24], anticryptococcus [25], antibacterial [26, 27], antitumor [28], anti-inflammatory [29, 30], antidiabetic [31], antioxidant [18], wound healing [32], and antihelmintic activities [33]. In recent times, the outbreak of drug-resistant diseases has led to several health issues. In an attempt to resolve these issues, pharmacological research has been tailored towards the discovery of innovative, potent, and safe drugs from natural compounds. This review appraises the ethnobotanical and pharmacological activities of S*. alata*, thus justifying diverse traditional applications of the plant.

## 2. Review Methodology

Relevant documents were obtained from major scientific catalogues such as Pubmed, Google Scholar, EBSCO, SciFinder, Scopus, Medline, and Science Direct. Many publications sites were queried to procure information on ethnobotanical description and pharmacological activities of *S. alata.*

## 3. Ethnobotanical Description

### 3.1. Description and Classification

*S. alata* (L.) Roxb. is a flowering shrub of the Fabaceae family. It has the name “candle bush” owing to the framework of its inflorescences [34]. It is an annual and occasionally biannual herb, with an average height of 1 to 4 m, burgeoning in sunlit and humid zones. The leaves are oblong, with 5 to 14 leaflet sets, robust petioles (2 to 3 mm), caduceus bracts (2 × 3 by 1 × 2 cm), and dense flowers (20 × 50 by 3 × 4 cm). Zygomorphic flowers have bright yellow color with 7 stamens and a pubertal ovary. The fruit exists as a 10 to 16 × 1.5 cm tetragonal pod, thick, flattened wings, brown when ripe with many diamond-shaped brown seeds. It is propagated by seeds and dispersed to about 1,500 m above the sea level [35, 36].

Taxonomically, *S. alata* is classified as kingdom: Plantae; order: Fabales; family: Fabaceae; subfamily: Caesalpinioideae; tribe: Cassieae; subtribe: Cassiinae; genus: Senna; species: *S. alata*.

### 3.2. Geographical Distribution

*S. alata* is widely distributed in Ghana, Brazil, Australia, Egypt, India, Somalia, Sri Lanka, and all over Africa [37]. It is an ornamental plant native to the Amazon Rainforest [38]. Like other *Senna* species, it is cultivated in humid and tropic regions of Africa, Asia, West Indices, Mexico, Australia, South America, the Caribbean Islands, Polynesia, Hawaii, Melanesia, and different parts of India [39]. In Philippines, Thailand, and Indonesia, this shrub is widely dispersed and is cultivated for medicinal purposes [40].

### 3.3. Ethnobotanical Uses

In Ayurvedic, Sinhala, Chinese, and African traditional medicine, various parts of *S. alata* have shown diverse therapeutic activities in diseases control. In northern Nigeria, the stem, leaf, and root decoctions is used in treatment of wound, skin and respiratory tract infection, burns, diarrhoea, and constipation [41, 42]. Also, in the south-western regions, leaf decoction serves as an antidote to body and abdominal pain, stress, and toothache [43]. It also cures dermal infections, convulsion, and as purgative [44, 45]. In Egypt, the leaf decoction was observed to be used as a bowel stimulant to stimulate peristaltic shrinkages and decrease water absorption from the colon in attempt to prevent constipation [46]. In Cameroon, the stem, bark, and leaves were reported to be used as a remedy for gastroenteritis, hepatitis, ringworm, and dermal infections [47].

In India, Philippines, and China, *S. alata*'s stem, bark, and leaf decoction were found to be effective in treating haemorrhoids, inguinal hernia, syphilis, intestinal parasitosis, and diabetes [48]. Currently, seeds and roots are used in regulating uterus disorder and worms [18, 49]. The leaves and flowers are typically used as antifungal agents and laxatives [35]. In China, the seeds are used in treating asthma and to improve visibility [50]. In Guatemela, Brazil, and Guinea, the whole plant is currently used in the treatment of flu and malaria [36]. The flower and leaves decoction was observed to be used in treatment of ringworms, scabies, blotch, eczema, scabies, and tine infections [11, 51]. The leaves are currently used in Sierra Leone to relieve abortion pain and facilitate baby delivery [51]. In west and east Africa, bark decoction is spread on cuts during tribal mark incision and tattoo making [52]. In China, India, Benin republic, Ghana, Nigeria, and Togo, the whole plant is used as a curative for Diabetes mellitus [34, 53]. Fresh leaves of the plant are used in the treatment of skin rashes, mycosis, and dermatitis [54]. From the traditional uses, the frequent use of *S. alata* leaves is more than that of roots, flowers, roots, and stem-bark owing to more active metabolites reported in the leaves. Besides its pharmacological activities, it is processed into capsule, pellet, and tea in Nigeria for preventing diseases and maintaining sound health.

### 3.4. Pharmacological Activities

**Figure 1:**
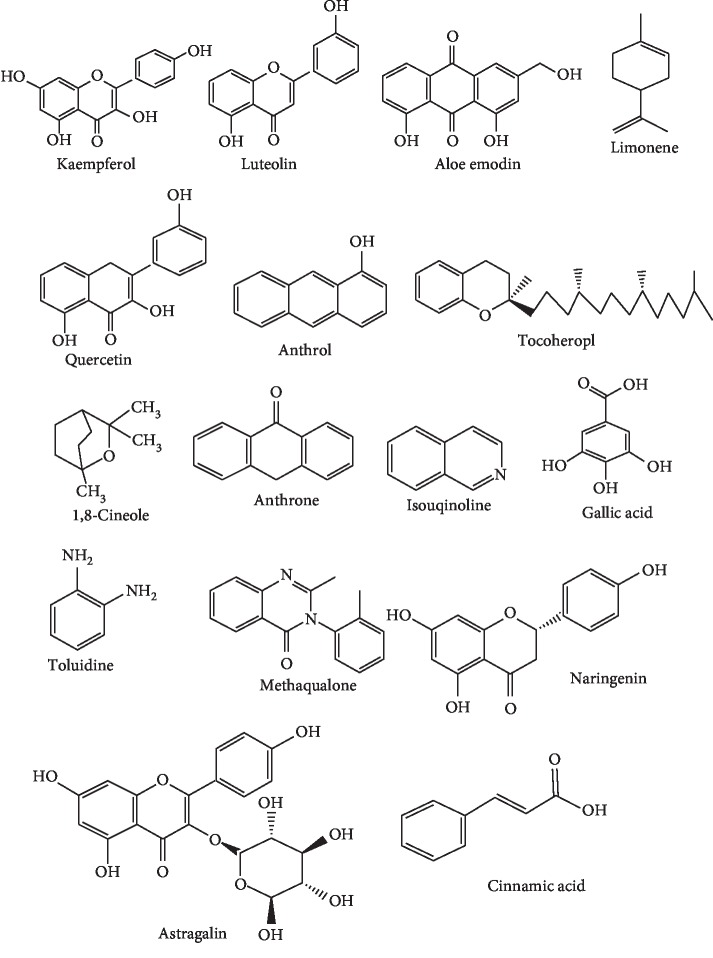
Bioactive compounds with therapeutic potencies in *S. alata*.

Medicinal plants belonging to the Fabaceae family have extensively been investigated for their pharmacological activities. Plants synthesised array of secondary metabolites which contribute to its therapeutic activities (Figure 1). Therapeutic appraisal of *S. alata* authenticates the ethnobiological claims and establishes the pharmacological activities (Table 1). There are many published articles connected to diverse therapeutic activities of *S. alata* which are mainly related to antibacterial, antidiabetic, antilipogenic, antifungal, antioxidant, dermatophytic, antihyperlipidemic, and anthelmintic activities among others. Few studies have also reported its antimalarial activities.

#### 3.4.1. Antibacterial Activities

The antibacterial potentials of medicinal herbs are appraised using zone of inhibition (ZOI) or minimum inhibitory concentration (MIC). The *in vivo* antibacterial potential of *S. alata* was assessed against methicillin-resistant *S. aureus* (MRSA), extended spectrum beta-lactamase, and carbapenemase-resistant. Enterobacteriaceae was isolated from infectious patients, via Mueller–Hinton broth via the microdilution technique. The extract showed significant activities at 512 mg/ml due to the flavonoids, quinones, tannins, sterols, alkaloids, and saponins analysed [75]. *S. alata* leaves collected from an Indian aboriginal tribe, displayed significant ZOI of 21 to 27 mm against clinical isolates of multidrug-resistant (MDR) bacteria, obtained from infected patients, when subjected to ASTs via Kirbye Bauer's disc diffusion assay (KBDD) [58].

Ciprofloxacin (30 *μ*g/disc) and vacuum liquid chromatographic (VLC) fractions of *S. alata* methanolic extract were assessed against *Staphylococcus aureus*, *Bacillus subtilis, Bacillus cereus* (Gram positive), *Shigella boydii, Shigella dysenteriae, Pseudomonas aureus, Vibrio mimicus*, *Salmonella paratyphi, Vibrio parahaemolyticus*, and *Salmonella typhi*, (Gram negative) using the disc diffusion assay. The fractions exhibited significant inhibitory activities against bacteria isolates at 100 *µ*g/ml [76].

Bioactive compounds such as kaempferol, luteolin, and aloe emodin isolated from the methanolic leaves extract was assessed against MDR bacteria strains. Aloe emodin exhibited strong MIC activities (4 to 128 *μ*g/ml). The marked activities could be linked to the anthraquinone and flavonoids compounds detected [59]. The antibacterial activity of chrysoeriol-7-O-(2″-O-beta-D-mannopyranosyl)-beta-D-allopyranoside,3-O-gentiobioside, quercetin, naringenin, and rhamnetin-3-O-(2″-O-beta-D-mannopyranosyl)-beta-D-allopyranoside isolated from chloroform, ethanol, petroleum ether, methanol, and aqueous leaves extract of *S. alata,* significantly inhibited the growth of *B. subtilis, V. cholerae, Streptococcus* sp., *S. aureus*, and *E. coli.* The extracts displayed pronounced activities which are in close proximity to penicillin, chloramphenicol, fluconazole, and ciprofloxacin [77].

Anthraquinone and flavonoid glycosides detected in *S. alata* extracts significantly inhibited the growth of *E. coli* and *S. aureus* with ZOI between 9.7 and 14.8 mm [22, 23]. The crude extracts and isolates obtained from purified fractions of *S. alata* flower extract (anthraquinone glycosides, steroids, tannins, and volatile oils) were assessed on selected bacteria isolates. Strong inhibitory activities were displayed at MIC of 500 mg/ml against the clinical isolates of *S. faecalis, B. subtilis, S. aureus, Pseudomonas putida*, and *M. Luteus.* At 500 mg/ml, ZOI of 10 to 25 mm was observed, with close proximity to the inhibitory activities displayed by streptomycin, penicillin, and methicillin [41]. The synergic effect of *Eugenia uniflora* and *S. alata* leaves was examined on clinical isolates of *B. subtilis* and *S. aureus.* The dried leaves were processed into a local antiseptic herbal soap and the antibacterial potential was assessed via hole-in-plate agar diffusion assay. The herbal soap significantly inhibited the growth of the tested organisms [78].

#### 3.4.2. Antioxidant Activities

The antioxidant or scavenging activities is important in appraisal of therapeutic potential of medicinal herbs. Plants played significant role in protecting cell against oxidative stress caused by active free radicals [79]. The free radical scavenging potentials of plants are appraised by antibiotic sensitivity tests (ASTs); 2,2-diphenyl-1-picrylhydrazyl (DPPH); ferric-reducing antioxidant power (FRAP); 2,2′-azino-bis(3-ethylbenzothiazoline-6-sulphonic acid) (ABTS); hydroxyl radical scavenging activity (HRSA), and metal ion chelating activity (MICA). The methanolic and n-hexane leaves extracts of *S. alata* were explored for their scavenging effects via DPPH assay. The methanolic extract displayed pronounced scavenging activities when compared to n-hexane extract. The methanolic extract was fractionated, leading to isolation of three major bioactive compounds (kaempferol, butylated hydroxytoluene, and emodin). The kaempferol fraction displayed pronounced scavenging activity than the butylated hydroxytoluene and emodin fractions [80]. Several bioactive compounds such as ascorbic acid, flavonoids, tocopherol, anthraquinone, and carotene assessed contributed to scavenging activities displayed [77].

Different parts of *S. alata*, significantly inhibited the action of free radicals causing oxidative stress [82]. The *in vitro* DPPH assay of ethyl acetate-DCM, methanol-DCM, and oil fractions of *S. alata* leaves was assessed using L-ascorbic acid as control. The radical scavenging activities measured at 517 nm significantly inhibited the free radicals at 500 g/ml. The concentration at 50% inhibition (IC_50_) of the fractions revealed activities of methanol-DCM (65.03%), ethyl acetate-DCM (62.33%), and oil fraction (64.10%). The strong scavenging activities could be due to polyphenol and flavonoid analysed [76].

#### 3.4.3. Antifungal Activities

Several bioactive compounds isolated from *S. alata* exhibit strong *in vitro* and *in vivo* antifungal activities. The antifungal activities of cannabinoid alkaloid (4-butylamine 10-methyl-6-hydroxy cannabinoid dronabinol), 1,8-cineole, caryophyllene, limonene, *α*-selinene, *β*-caryophyllene, germacrene D, hexadecanoic acid methyl ester, hexadecanoic acid, (6Z)-7,11-dimethyl-3-methylidenedodeca-1,6,10-triene, octadecanoic acid methyl ester, cinnamic acid, 3,7-dimethylocta-1,6-diene, pyrazol-5-ol, flavonol and gallic acid, methaqualone, and isoquinoline have been explored [51,83–85].

Volatile oils extracted from *S. alata* flowers were assessed against standard strains and clinical isolates of *Candida* and *Aspergillus* species. The oils significantly inhibited the growth of the tested microbes [86]. The methanolic extract and purified n-hexane and ethanolic fractions of *S. alata* flower displayed strong inhibitory activities against *A. Niger, C. utilis, G. candidum, A. brevipes*, and *Penicillium* species with an MIC of 0.312 to 5 mg/ml. However, at different concentrations, the purified fractions exhibited prominent inhibitory activities than the methanolic extracts. Likewise, mycelia growth was significantly inhibited by the purified fractions, with a total suppression of sporulation for 96 h at 2 mg/ml, compared to less sporulation after 48 h for methanolic extracts [87].

The aqueous and ethanolic leaf and bark extracts of *S. alata* was assessed via disc diffusion assay against *M. canis*, *C. albicans*, and *A. fumigatus.* The bark extracts displayed prominent concentration-dependent susceptibility against *C. albicans.* On the contrary, the aqueous extract showed a strong ZOI of 12 and 16 mm, while ethanolic extracts exhibited 10 and 14 mm. However, tioconazole displayed strong inhibitory activities of 18 mm which are significantly higher than the extracts at equivalent concentration [64].

*S. alata* flowers and leaves collected from Ogbomoso, Southwest Nigeria, were examined in an attempt to justify the indigenous claims of its antifungal efficacy. *In vitro* antifungal activities of ethanolic and methanolic extracts were investigated using the disc diffusion approach. Ethanolic extracts showed pronounced inhibitory activities when compared to methanolic extracts. The IC_50_ value of the ethanolic extract was two folds higher than the methanolic extracts against the fungi isolates. The inhibitory activities displayed could be due to methaqualone, cinnamic acid, isoquinoline, and toluidine detected [71].

#### 3.4.4. Dermatophytic Activities

Currently, the leaves, flowers, and bark of *S. alata* are used for treating various kinds of skin infections and diseases. In Thailand, the plant was mentioned as one of the 54 medicinal plants used for treating scabies, shingles, urticarial, itching, pityriasis versicolor, and ringworm [88]. The dermatophytic activities displayed by *S. alata* are linked to the bioactive compounds such as anthranols, anthrones, flavonoids, phenols, tannins, and anthracene derivatives [89]. Leaves decoction displayed strong inhibitory activities against *S. pyogenes, S. aureus, K. pneurnoniae, E. coli, S. rnarcescens, P. cepacia,* and *P. aeruginosa* [90, 91].

Cannabinoid alkaloid (4-butylamine 10-methyl-6-hydroxy cannabinoid dronabinol) and apigenin isolated from *S. alata* seeds were incorporated in a local antiseptic soap. The soap significantly hindered the spread of ringworms, eczemas, carbuncles, boils, infantile impetigo, and breast abscess [84]. The leaf, stem-bark, flower exudates, and ethanolic leaf extract examined against clinical isolates of *T. jirrucosum, M. canis, T. mentagrophyte, E. jlorrcosum, B. dermatitidis, A. flavus,* and *C. albicans* displayed strong inhibitory activities against the causative organisms [18,92–94].

#### 3.4.5. Antilipogenic, Antidiabetic, and Antihyperlipidemic Activities

In Africa and Asia, *S. alata* leaves and flowers are currently used to regulate lipid absorption, obesity, and fat levels in blood serum. Aqueous leaf extract considerably reduced blood glucose levels, serum cholesterol, triglyceride, hepatic triglyceride, serum leptin, and insulin levels in Wistar mice [57].

Diabetes is a threat to man leading to stern socio-economic and high rate of mortality [95]. Herbal decoction of *S. alata* is commonly used in Caribbean [96], Africa [34], and India [97] for the treatment of diabetes. The marked *α*-glucosidase inhibitory effect and antidiabetic activities displayed by *S. alata* are linked to bioactive compounds such as astragalin and kaempferol-3-O-gentiobioside [31, 98]. Traditionally, the leaves decoction is used in regulation of sugar level in serum [99] and enhances the defence of hepato and renal organs to oxidative stress [56]. It also reduces fasting blood glucose and improves metabolic state via activation of PKB/Akt which helps regulate glucose uptake in the liver and muscles [100].

The antidiabetic potency of acarbose, n-butanol, and ethyl acetate fractions of methanolic *S. alata* leaf extract was assessed via inhibitory carbohydrate digestion mechanism. The antidiabetic drug (acarbose) significantly inhibits *α*-glucosidase with inhibitory concentration (IC_50_, 107.31 ± 12.31 *µ*g/ml). n-Butanol and ethyl acetate fractions displayed inhibitory activities of IC_50_, 25.80 ± 2.01 *µ*g/ml and IC_50_, 2.95 ± 0.47 *µ*g/ml, respectively, due to kaempferol 3-O-gentiobioside analysed by HPLC and Combiflash chromatography [31, 83]. *α*-Amylase and *α*-glucosidase inhibitory potential of acetone, hexane, and ethyl acetate leaf extracts of *S. alata* was assessed via Lineweaver–Burk plot. Acetone extract significantly inhibited *α*-amylase (IC_50_ = 6.41 mg/mL), while hexane displayed strong inhibition against *α*-glucosidase (IC_50_ = 0.85 mg/mL). n-Hexane substantially reduces (*p* < 0.05) postprandial blood glucose in sucrose-induced rats after 2 h of treatment [101].

The reduction in the oxidative stress in the aorta and heart of streptozotoc, hyperglycemic rats was investigated by DPPH free radical scavenging and antioxidant catalase assays. The aqueous leaf extract significantly reduced lipid peroxidation (MDA levels), blood glucose; however, it increases in the antioxidant catalase and DPPH free radical scavenging activity was observed. The reversal of oxidative stress induced by cardiac dysfunction in hyperglycemic condition justified the ethnobiological claim of the plant [102]. In a similar study, the *S. alata* leaf extract significantly reduces oxidative stress causing diabetes in treated mice. Prominent activities were observed in the kidney and liver. The activity resulted in critical changes in the urea, protein, creatinine, and uric acid level in the blood serum [56].

#### 3.4.6. Antimalarial Activities

Malaria is a global threat contributing to serious sociocultural and health issues in humid and tropical regions [103, 104]. Chemotherapy reported the antimalarial activities of *S. alata* could be linked to quinones, alkaloids, and terpenes [105, 106]. Quinones isolated from *S. alata* significantly displayed *in vitro* antiplasmodial activity, against *Plasmodium falciparum* via the microdilution test of Desjardin [107]. Terpenes isolated from *S. alata* leaves displayed pronounce *in vitro* antiplasmodial assays against *P. falciparum* in ethylene glycol-water fractions. Significant activity was observed at concentration below 1 *µ*g/ml [108]. The appraisal of aqueous leaf extract justifies the ethnomedical applications of *S. alata* as remedy for malaria and fever. The leaf extract considerably inhibited 3D7 strain of the *P. falciparum* parasite in Wistar mice [109].

#### 3.4.7. Anthelmintic Activity

Traditionally, *S. alata* leaf and flower decoctions are used in treatment of intestinal worm infestation and stomach disorder. The anthelmintic potency of alcoholic leaves extract of *S. alata* and *T. angustifolia* at 10 to 100 mg/ml were assessed in clinical isolates of *Ascardia galli* and *Pheretima posthuma* by observing time of paralysis and point of death of the worms. The leaves extract significantly inhibit the worms (test organisms) more than piperazine citrate (standard anthelmintic drug) [110].

#### 3.4.8. Antiviral Activities

*S. alata* is an indispensable bactericidal and fungicidal natural therapy. However, the justification of antiviral activities is not properly documented. The antiviral efficacy of n-hexane, ethyl acetate, butanol, and aqueous leaf extracts was assessed on dengue virus (DENV) obtained from an infected pregnant woman in Indonesia via focus assay. The extracts significantly inhibited DENV-2 with IC_50_ (<10 *μ*g/ml), CC_50_ (645.8 *μ*g/ml) and SI (64.5 *μ*g/ml) [111].

## 4. Nutritional Constituents of *S. alata*

In appraisal of the therapeutic potentials of medicinal herbs, phytochemical, pharmacological, toxicological, and nutritional details are indispensable. In appraisal of the nutritional constituents of *S. alata* leaf, several nutrients assessed include moisture content (9.53 ± 0.06), carbohydrate (298.61 ± 0.40), crude lipid (47.73 ± 0.01), crude fibre (18.23 ± 0.13), and ash (15.73 ± 0.03). The nutritional contents analysed in flower revealed ash (7.00 ± 1.0), crude protein (13.14 ± 0.02), carbohydrate (57.04 ± 0.04), moisture (6.16 ± 0.14), and crude lipid (1.81 ± 0.09). It is observed that storage capacity, easy absorption of food, antimicrobial and anaesthetic properties are connected to the nutritional composition of herbs [112]. Several minerals such as potassium, iron, manganese, calcium, zinc, copper, and chromium have been assessed in *S. alata.* These mineral constituents help in formulation of herbal drugs and dietary supplements [113–115].

## 5. Toxicological Studies of *S. alata*

Lately, medicinal plants and herbal products have come under spotlight owing to health hazards connected to indiscriminate handling of herbs and herbal products. Several reports were published relating to the use of herbal drugs. Despite the risks ascribed to most medicinal and herbal products, the number of herbal users still increases. This initiates the appraisal of herbs in an attempt to establish their safety [116]. The *S. alata* leaf extract was appraised by oral administration of Wistar rats (10 g/kg weight). The acute and subacute effects were assessed in every 24 h for 14 days at different dosages (250, 500, and 1000 mg/kg). After 24 h, no significant variation was observed in the liver, urea, bicarbonate, creatinine, and kidney of the tested organisms and control [117]. Aqueous flower extract at doses of 100, 400, and 800 mg/kg was orally administered to ten Wistar rats for 28 days. No significant difference on histological sections of the lung, liver, kidney, heart, and kidney was observed during the postmortem analysis of the mice [118].

Different doses (1,000, 2,000, and 3,000 mg/kg body weight) of ethanolic leaf extract do not display any behavioural, biochemical, haematological, and histopathological variations in the tested Swiss albino mice and control after 15 days of oral administration [119]. Hydroethanolic leaf extract increased the body weight, blood level, liver homogenates, and serum index after proper monitoring of the administered rats every 48 h for 26 days. Haematological parameters assessed displayed dose-dependent response, however, no significant variation was observed in liver histopathology [120]. Alkaloids isolated from aqueous *S. alata* leaf extract significantly reduces alkaline phosphate, uric acid, globulin, liver and kidney body weight ratio, urea, phosphate ions, bilirubin, potassium ions, serum concentrations of albumin, creatinine, gamma glutamyl transferase, alanine transaminases, and aspartate in the liver and kidney of pregnant rats after periodic observation for 18 days. This led to variation in serum enzymes, excretory, and secretory functions of liver and kidney of the test organisms [61].

Documented toxicological appraisals of *S. alata* present positive information regarding its safety; however, there is need to authenticate these reports through *in vitro* and *in vivo* assessments on clinical isolate. Fresh and dried plant parts, low-dosage concentrations, test organisms of different age groups and effect on other vital organs such as brain, intestine, and bladder should be considered during toxicological appraisals of medicinal plant or products. Ethnobiological appraisals described that ulcer patients, pregnant women, and children under the age of two should avoid *S. alata* decoction. Also, studies should be focused on appraisals of recommended dosage, safest mode of administration, and reaction mechanisms of the metabolites in living organisms.

## 6. Limitation, Conclusion, and Future Prospects

Based on this review, further studies on *S. alata* have become evident. Most studies focused on lipid-soluble and volatile components; however, appraisals on metabolites prepared in hydrophilic medium are deficient. Most phytochemical studies of *S. alata* focused on leaves, stems, and flowers, though study on seeds and roots are lacking. Many therapeutic appraisals have been investigated in cell and animal experiments; still, there remain important tasks. These include secondary metabolites dictating the pharmacological activities are not well stated; relatively, few studies analytically appraised the mechanisms of action of secondary metabolites; systemic toxicological studies remain ambiguous. The phytochemical appraisals revealed there is a variation in some metabolites investigated due to geographical and climatic factors. This gives room for qualitative approach to authenticate the metabolites in this plant. The plant secondary metabolites contributed to the pronounced wound healing and antibacterial and antifungal activities [92, 121]. Industrially, the plant is a key ingredient in detergents (foaming and surface active agents), molluscicides and pesticides. Summarily, comprehensive ethnobiological and pharmacological activities of *S. alata*, limitations, and future prospects were explored which could present novel research directions.

## Figures and Tables

**Table 1:** Pharmacological activities of *S. alata*.

	Parts used	Country	Ethnomedicinal use	Solvent used	Pharmacological activity	Model used	Phytochemicals	References
(1)	Leaves	Nigeria	Treatment of diarrhoea, upper respiratory tract infection, and to hasten labour	Aqueous	Abortifacient	Pregnant rats	Saponins, flavonoids, cardiac glycosides, cardenolides, dienolides, phenolics and alkaloids	[55]
(2)	Leaves	India	To manage diabetes	Ethanolic	Hepato-renal protective effects	Male albino Wistar rats		[56]
(3)	Leaves	India	Treatment of allergy/asthma	Hydromethanolic	Antiallergic	Lipoxygenase (LOX) enzyme	Rhein and kaempferol	[7]
(4)	Leaves	Thailand	To manage diabetes and weight	Aqueous	Antilipogenic	High-fat diet-induced obese mice		[57]
(5)	Leaves	India	Treatment of bacterial infections	Methanol	Antibacterial	Pathogenic bacterial strains		[58]
(6)	Leaves	Cameroon	Treatment of gonorrhoea, gastrointestinal and skin diseases	Methanol	Antibacterial	Multidrug-resistant (MDR) *Vibrio cholerae* and *Shigella flexneri*	Kaempferol, luteolin and aloe-emodin	[59]
(7)	Leaves	India	To manage diabetes	Methanol	Antidiabetic	*α*-Glucosidase enzyme	Kaempferol 3-O-gentiobioside	[31]
(8)	Leaves	Thailand	Treatment of skin infections	Anthraquinone	Antifungal	STZ-induced diabetic rats	Anthraquinone	[60]
(9)	Leaves	Nigeria	To wash the uterus	Hexane	Anti-implantation, antigonadotropic, antiprogesteronic	Ovariectomized female rats	Alkaloids	[61]
(10)	Flower	Nigeria	Treatment of urinary tract infections and gonorrhoea	Methanol	Antibacterial	Bacterial strains	Steroids, anthraquinone glycosides, volatile oils and tannin	[62]
(11)	Flower	India	To treat scabies and ringworm	Aqueous	Antifungal	Aflatoxin producing and human pathogenic fungi		[63]
(12)	Leaves and barks	Malaysia	To treat superficial fungal infections	Ethanol and water	Antimicrobial	Bacterial and fungal strains		[64]
(13)	Leaves	Cameroon		Ethanol	Antioxidant and anti-inflammatory	White blood cells		[30]
(14)	Leaves	India	Used as purgative expectorant, astringent, vermicide	Ethanol	Anticancer	Male Wistar rats	Alkaloid, flavonoids, saponins, tannins glycosides	[65]
(15)	Leaves	Indonesia	Treatment of malaria; antioxidant and antibacterial	Ethanol	Antiviral	DENV-2 and Huh 7it cells	Flavonoid	[66]
(16)	Petals	India	As an immune stimulant	Petroleum ether	Immunomodulatory	*Garra rufa* fish	Cardiac glycosides, phenols, anthraquinone, alkaloids	[67]
(17)	Root	Nigeria	As an abortifacient in women, for the termination of early pregnancy	Ethanol	Uterine smooth muscle	Male and female Albino mice	Alkaloids	[68]
(18)	Leaves	Burkina Faso	Treatment of asthma-induced bronchospasm	Aqueous and ethanolic	Bronchorelaxant, genotoxic, and antigenotoxic	Male and female Wistar rats		[69]
(19)	Leaves	Egypt	Treatment of skin tumour	Methanol	Antitumor	Human cancer cell lines (HepG2, MDA-MB-231, and Caco2)	Palmitic, linolenic, linoleic, stearic acid	[70]
(20)	Leaves	Thailand	Laxative	Methanol	Anti-inflammatory	Tert-butyl hydroperoxide-induced oxidative stress in HaCaT cells	Rhein	[71]
(21)	Leaves, flower and fruit	Nigeria	Laxative and treatment of microbial infections	Methanol and ethanol	Antifungal and antibacterial	Clinical bacterial and fungal isolates	Flavonoids	[72]
(22)	Leaves	Thailand	In regulating glucose level in the blood	Aqueous	Antilipogenic	Male ICR mice		[73]
(23)	Leaves	Cameroon	To cure fever	Aqueous and methanolic	Antiplasmodial	RPMI 1640 and albumax		[74]

## Abbreviations

ZOI: Zone of inhibition
MIC: Minimum inhibitory concentration
MDR: Multidrug resistant
ASTs: Antibiotic sensitivity tests
DPPH: 2,2-Diphenyl-1-picrylhydrazyl
FRAP: Ferric-reducing antioxidant power
ABTS: Chemiluminescence, 2,2′-azino-bis(3-ethylbenzothiazoline-6-sulphonic acid)
HRSA: Hydroxyl radical scavenging activity
MICA: Metal ion chelating activity.
